# Different Effects of Implicit and Explicit Motor Sequence Learning on Latency of Motor Evoked Potential Evoked by Transcranial Magnetic Stimulation on the Primary Motor Cortex

**DOI:** 10.3389/fnhum.2016.00671

**Published:** 2017-01-04

**Authors:** Masato Hirano, Shinji Kubota, Yoshiki Koizume, Shinya Tanaka, Kozo Funase

**Affiliations:** ^1^Human Motor Control Laboratory, Graduate School of Integrated Arts and Sciences, Hiroshima UniversityHiroshima, Japan; ^2^Japan Society for the Promotion of ScienceTokyo, Japan

**Keywords:** implicit learning, explicit learning, TMS, MEP latency, motor cortex

## Abstract

Motor training induces plastic changes in the primary motor cortex (M1). However, it is unclear whether and how the latency of motor-evoked potentials (MEP) and MEP amplitude are affected by implicit and/or explicit motor learning. Here, we investigated the changes in M1 excitability and MEP latency induced by implicit and explicit motor learning. The subjects performed a serial reaction time task (SRTT) with their five fingers. In this task, visual cues were lit up sequentially along with a predetermined order. Through training, the subjects learned the order of sequence implicitly and explicitly. Before and after the SRTT, we recorded MEP at 25 stimulation points around the hot spot for the flexor pollicis brevis (FPB) muscle. Although no changes in MEP amplitude were observed in either session, we found increases in MEP latency and changes in histogram of MEP latency after implicit learning. Our results suggest that reorganization across the motor cortices occurs during the acquisition of implicit knowledge. In contrast, acquisition of explicit knowledge does not appear to induce the reorganization based on the measures we recorded. The fact that the above mentioned increases in MEP latency occurred without any alterations in MEP amplitude suggests that learning has different effects on different physiological signals. In conclusion, our results propose that analyzing a combination of some indices of M1 excitability, such as MEP amplitude and MEP latency, is encouraged in order to understand plasticity across motor cortices.

## Introduction

We acquire many motor skills in everyday life. It is well known that there are two types of learning process (Willingham, 1998, 2001). The one is implicit learning in which a new motor skill is acquired with a little awareness, and the other is explicit learning that we intentionally learn a new skill. The primary motor cortex (M1) plays a critical role in the development of implicit knowledge (Nitsche et al., 2003; Robertson et al., 2005; Kantak et al., 2012). A previous study demonstrated increases in the M1 excitability during the acquisition of implicit knowledge (Pascual-Leone et al., 1994). However, a recent study showed that the amplitudes of the motor evoked potentials (MEP) evoked by transcranial magnetic stimulation (TMS) did not change during implicit learning, whereas they decreased during explicit learning (Tunovic et al., 2014). Increases and decreases in the amplitudes of MEP are often considered to represent evidence of long-term potentiation (LTP) and long-term depression (LTD), respectively, and a lack of such changes is considered to be evidence of the absence of plasticity (Thickbroom, 2007). Therefore, the contradictory results obtained in the previous two studies have resulted in confusion regarding the plasticity of the M1 during the acquisition of implicit and explicit knowledge.

In earlier studies, researchers often focused on the changes in MEP amplitude through motor learning (Pascual-Leone et al., 1994; Tunovic et al., 2014). However, there are three situations in which a lack of variation in MEP amplitude does not reflect the absence of plasticity. Specifically, MEP amplitude does not change when: (1) excitatory and inhibitory plasticity (e.g., LTP, LTD, synaptogenesis and synapse elimination) are balanced at the stimulation site; (2) the plastic changes induced in the M1 by motor learning occur around the stimulation site; and (3) the changes in the excitability of the M1 are canceled out by changes in spinal excitability. In the present study we focused on MEP latency, which provides meaningful information about the corticospinal tract, as well as MEP amplitude, in order to examine learning-induced plasticity. TMS induces I-waves, which result from indirect activation of corticospinal neurons via cortical interneurons. Therefore, MEP latency is considered to reflect the composition of I-waves (Day et al., 1989; Sakai et al., 1997). We expected that MEP latency would change along with the reorganization of the M1 even in cases in which excitatory and inhibitory plasticity canceled each other out because the plastic changes in the neural circuits of the M1 would alter the signaling responsible for inducing changes in the composition of I-waves. Another possibility is changes in intrinsic property of early I-wave component through motor learning. For example, changes in rising time of EPSP induced by early I-wave component would alter MEP latency without changes in MEP amplitude.

Here, we investigated the reorganization of the M1 brought about by implicit or explicit learning during a serial reaction time task (SRTT) by measuring the amplitude and latency of the MEP with a mapping procedure.

## Materials and Methods

### Subjects

Twenty one healthy right-handed subjects with no experience of specific musical instrument training participated in this study after giving their written informed consent (6 females, mean age: 23.1 ± 1.22). Seventeen participants (5 females, mean age: 23.0 ± 1.22) joined in a first experiment, and eight participants (1 female, mean age: 23.4 ± 1.51) joined in an additional experiment (four subjects also joined in first experiment; interval between the two experiments was at least 1 year). In the first experiment, we designed the experiment as a cross-over study involving two sessions. Each session was separated by at least 1 week. All experimental procedures were carried out in accordance with the Declaration of Helsinki and were approved by the ethics committee of Hiroshima University, Japan. Vulnerable populations were not involved in this study.

### First Experiment

#### Serial Reaction Time Task

Based on the methods of previous studies (Robertson et al., 2004; Tunovic et al., 2014), our subjects performed a modified version of the SRTT. Five LED were arranged in a horizontal line in front of the subjects as a visual cue. We asked the subjects to press the appropriate key on a piano-type keyboard as fast as possible when an LED lit up (thumb = 1, index finger = 2, middle finger = 3, ring finger = 4, little finger = 5). When the subjects pressed the correct key, the LED was extinguished, and the next LED lit up after 400 ms.

In the implicit session, the subjects were told that the LED would light up randomly throughout the experiment so the subjects did not have any information about the sequence (2-3-1-4-5-3-2-5-4-1-3-5-4-2-1). In the explicit session, we told the subjects that the LED would light up sequentially (2-5-1-3-4-2-1-5-4-2-3-5-4-3-1) after the color of the LED changed from red to blue. The subjects were only told about the existence of the sequence, not its length or order. Nine subjects initially participated in the implicit session, whereas the other eight were subjected to the explicit session first.

The protocol and the number of trials were identical to those used in previous studies (Galea et al., 2010; Tunovic et al., 2014). Fifty random trials were performed before and after the sequential trials in both the training and test blocks. The training block was sandwiched between two test blocks, and subjects performed a test block 4 h after from the post-test block as a retention test. In the implicit session, the training block included 25 repetitions of the sequence, and the test block consisted of 15 repetitions of the sequence. In the explicit session, the training block consisted of 15 repetitions of the sequence, and the test block involved nine repetitions of the sequence. A learning rate during explicit learning was faster than that during implicit learning. If the number of sequence trial was the same between the implicit and explicit session, the amount of shortening of reaction time (RT) through training would markedly differ between the two sessions. Furthermore, it has been demonstrated that implicit knowledge is acquired in parallel with explicit knowledge (Willingham and Goedert-Eschmann, 1999). To minimize the effect of development of implicit knowledge on the results in explicit session, we reduce the number of trials in the explicit session.

For the performance measure, the mean RT during the sequence trials was calculated using all of the data within each block (i.e., 15 × 15 trials in the implicit session and 15 × 9 trials in the explicit session). The mean RT during the random trials was calculated using the last 50 trials within each block. For the explicit learning measure, we asked the subjects to recall items of the sequence that subjects memorized during the SRTT after the retention test. We counted the number of items of the sequence that the subjects correctly recalled. We excluded the subjects who noticed the existence of a sequence during the implicit training period and were able to recall more than four items of the 15-item sequence at the end of the experiment. Five subjects were excluded from the subsequent analysis (two subjects who initially participated in the implicit session and three who were subjected to the explicit session first).

### Transcranial Magnetic Stimulation and Electromyography

Surface electromyogram (EMG) recordings were obtained from the right-flexor pollicis brevis (FPB) muscle using Ag/AgCl surface electrodes. The EMG signals were amplified, filtered (5–3 kHz), and sampled at 10 kHz. A figure-of-8 coil with a diameter of 70 mm connected to a MagStim200 stimulator (Magstim, Whitland, UK) was used to evoke MEP in the FPB. We marked 25 stimulation sites around the optimal position (the hot spot) for evoking MEP on a cap worn by the subjects (Figure 3A). The hot spot was located centrally relative to the other stimulation sites, and the distance between each site was 1 cm. The resting motor threshold (rMT) was defined as the lowest intensity that evoked MEP of 50 μV in 5 out of 10 trials at the hot spot. We stimulated the M1 with a TMS intensity of 1.4 rMT at a rate of 0.2 Hz. The TMS coil was fixed tangentially to the scalp with the handle pointing backward and rotated ~45° away from the mid-sagittal line. Ten TMS pulses were applied at each of the 25 stimulation sites. We removed the MEP data from analysis if pre-trigger EMG was detected. MEP were recorded before the pre-test block and immediately after the post-test block. Each MEP collection period lasted approximately 25 min.

MEP latency was automatically calculated using a custom-made program written in Matlab (Mathworks, MA, USA). MEP onset was defined as a deviation from the mean plus 2*standard deviation (SD) value for the rectified EMG activity recorded before the TMS trigger. We removed the data for signals involving MEP amplitudes of <0.25 mV to ensure that MEP onset was detected in a stable manner. One subject (who participated in the implicit session first) was excluded because we could not calculate MEP latency due to a large TMS artifact on their EMG recordings. We compared the relative frequency of MEP latency, which consists of MEP obtained from all 25 sites, between the pre-training and post-training periods. We normalized MEP latency using a shortest MEP latency recorded during the pre-training period to examine the changes in MEP latency across subjects because mean MEP latency differed across subjects. First, we defined a shortest MEP latency as a bin which the relative frequency of MEP latency in the pre-training period was firstly above 1%. Then, other MEP latencies were subtracted with the shortest MEP latency. This procedure allowed us to calculate the mean MEP histogram across subjects. We showed the range of the normalized MEP latency from 0 ms to 4 ms because there were few MEP that exhibited normalized MEP latency of >4 ms.

### Additional Experiment

To examine the effects of different trial number of SRTT between the implicit and explicit session on the results of first experiment, we performed an additional experiment. In this experiment, subjects performed the SRTT with explicit manner. The training block included 25 repetitions of the sequence, and the test block consisted of 15 repetitions of the sequence. Before and after the training, we recorded MEP from FPB muscle evoked by TMS on the 25 stimulating points as with the first experiment.

### Statistical Analysis

Three-way repeated measures analysis of variance (ANOVA) was used to test the differences in the MEP amplitude in each site (session [implicit, explicit] × time [pre, post] × site [25 sites]), and the differences in histogram of the MEP latency (session [implicit, explicit] × time [pre, post] × latency [0–4 ms]), and the differences in the histogram of the MEP amplitude (session [implicit, explicit] × time [pre, post] × amplitude [0.3–5 mV]). Two-way repeated measures ANOVA was also used to test the differences in amount of skill improvement between the implicit and explicit session (session [implicit, explicit] × time [pre, post]), and the mean MEP latency (session [implicit, explicit] × time [pre, post]). Bonferroni’s* post hoc* test was used to further the analysis. Mauchly’s test examined sphericity in the repeated measures ANOVA models. Mauchly’s test of sphericity was not significant in all repeated measures ANOVA’s test. We calculated point-biserial correlation coefficient (*r*) and partial *η*^2^ as an effect size for the *post hoc* test and for the ANOVA, respectively. In all analyses, the level of statistical significance was set at *p* < 0.05. All data are shown as the mean ± SE values.

## Results

### First Experiment

**Figure 1:**
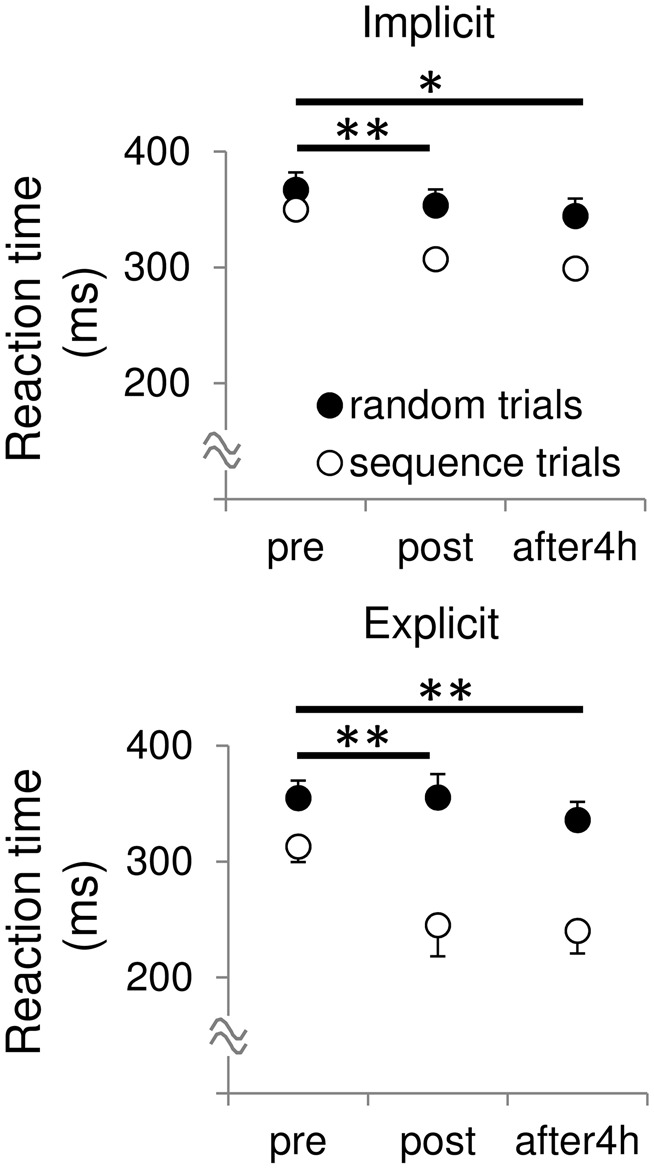
**Mean reaction times (RT) during the sequential and random trials before, after and 4 h after the training in both the sessions.** All data are represented as mean ± SE values. skill, pre vs. post, **p < 0.01, *p < 0.05.

We calculated the difference in mean RTs between the random and sequence trials as a skill to analyze sequence specific improvement of RTs through the training. In both the sessions, Bonferroni’s *t*-test revealed a significant differences in skill between the pre- and post-test (Figure 1, implicit: *t* = 4.41, *p* < 0.01, *r* = 0.81; explicit: *t* = 3.94, *p* < 0.01, *r* = 0.78), and between the pre- and retention-test (implicit: *t* = 2.85, *p* < 0.05, *r* = 0.67; explicit: *t* = 3.53, *p* < 0.01, *r* = 0.75), but not between the post- and retention-test (implicit: *t* = 0.14, *p* = 0.89, *r* = 0.04; explicit: *t* = 1.61, *p* = 0.14, *r* = 0.45). To examine whether amount of skill improvement differed between the two sessions, we subtracted the post- and retention skill with pre-skill (i.e., post-skill—pre-skill, retention skill—pre-skill). Two-way repeated measures ANOVA yielded no significant main effect of session (*F*_(1,10)_ = 3.67, *p* = 0.08, partial *η*^2^ = 0.27), time (*F*_(1,10)_ = 1.44, *p* = 0.26, partial *η*^2^ = 0.13), and their interaction (*F*_(1,10)_ = 1.39, *p* = 0.27, partial *η*^2^ = 0.12) on the amount of skill improvement. This indicates that the amount of skill improvement did not differ between the two sessions.

For the explicit measure, we asked the subjects to recall the sequence at the end of the experiment and recorded the number of correctly recalled items. The subjects were able to recall 0.27 ± 0.27 items of the sequence in the implicit session. This indicates that the subjects learned the sequence implicitly through the implicit session. By contrast, the subjects were able to recall 8.82 ± 1.74 items of the sequence in the explicit session. Therefore, subjects learned the sequence explicitly through the explicit session.

**Figure 2:**
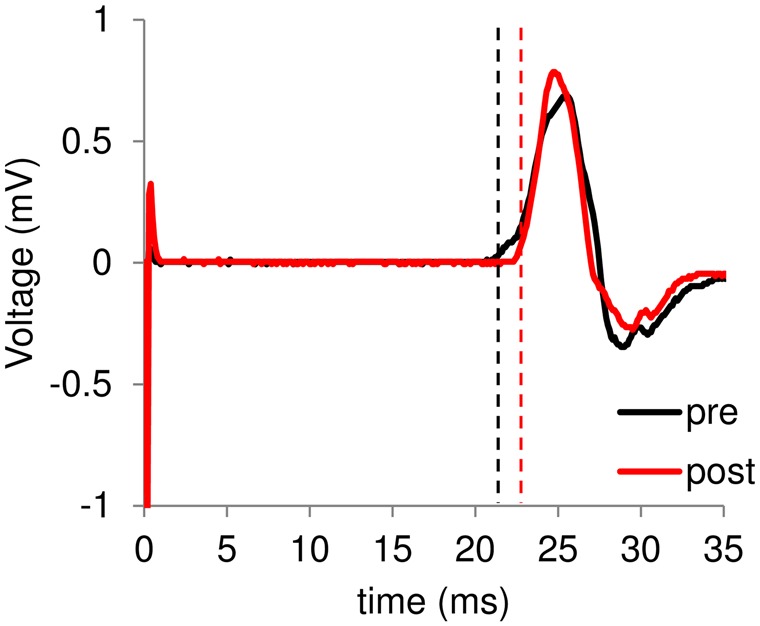
**A typical example of motor-evoked potentials (MEP) waveform.** Black and red lines represent electromyogram (EMG) traces obtained at the pre- and post-training session, respectively. In the horizontal bar, 0 ms means the time of transcranial magnetic stimulation (TMS) stimulation. Dashed lines represent onset of each MEP.

**Figure 3:**
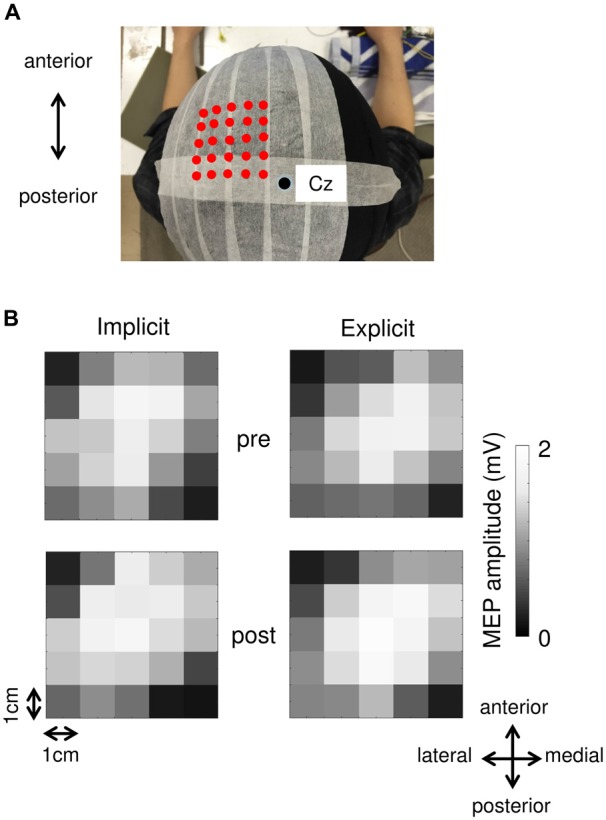
**(A)** An example of each stimulation site. Red dots indicate each stimulation site. A black circle represents the Cz. **(B)** MEP maps obtained at pre- and post-session in both the implicit and explicit sessions.

**Figure 4:**
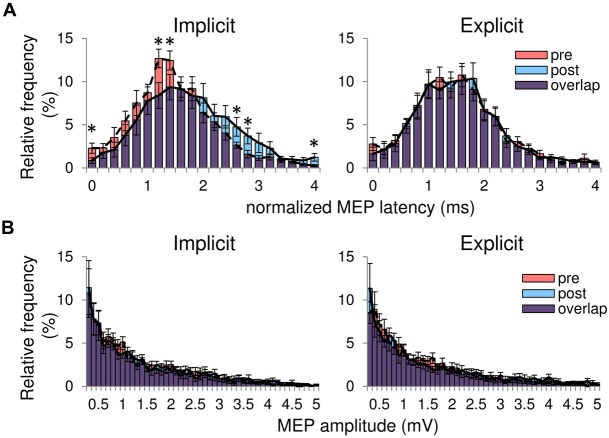
**(A)** Histograms of all MEP latency averaged from the 25 sites in both sessions. The horizontal axis represents the normalized MEP latency showing the difference in MEP latency between the shortest MEP latency recorded before the training and other MEP latency for each subject. The red bars are the pre-training histograms, and the blue bars are the post-training histograms. The purple bars represent overlapping areas. The dashed line connects the peak of each bin in the pre-training histogram. The solid line represents the peak of each bin in the post-training histogram. **(B)** Histograms of MEP amplitude in both sessions. All data are represented as mean ± SE values. *pre vs. post, *p* < 0.05.

Figure 2; Table 1 shows typical examples of MEP waveform and the summary of MEP measurements, respectively. There were no significant differences in the resting threshold, the root mean squared EMG (rmsEMG) of the pre-trigger EMG (100 ms prior to each TMS pulse), the mean MEP amplitude between the pre- and post-training, and between the implicit and explicit session. Furthermore, we examined differences in MEP amplitude between pre- and post-training in each stimulating site (Figure 3B). Three-way repeated measures ANOVA (session × time × site) yielded significant main effect of site (*F*_(24,240)_ = 10.17, *p* < 0.05, partial *η*^2^ = 0.50) on MEP amplitude obtained from the 25 sites, but there were no significant main effect of session (*F*_(1,10)_ = 0.54, *p* = 0.46, partial *η*^2^ = 0.05) and time (*F*_(1,10)_ = 3.13, *p* = 0.08, partial *η*^2^ = 0.24), and no significant first and second interaction between these factors (all *p* > 0.05). As for the factors affecting the mean MEP latency, two-way repeated measures ANOVA detected no significant main effect of time (*F*_(1,10)_ = 4.50, *p* = 0.06, partial *η*^2^ = 0.31) and session (*F*_(1,10)_ = 1.46, *p* = 0.26, partial *η*^2^ = 0.13), but the interaction between these parameters was significant (*F*_(1,10)_ = 5.10, *p* < 0.05, partial *η*^2^ = 0.34). Bonferroni’s *post hoc* test detected a significant increase in MEP latency in the implicit session (Table 1: *t* = 2.52, *p* < 0.05, *r* = 0.62), but not the explicit session (*t* = 0.79, *p* = 0.45, *r* = 0.24). To reveal the details of the changes in MEP latency, we compared the relative frequency of MEP latency between the pre-training and post-training periods. To calculate the mean histogram of MEP latency across subjects, we normalized MEP latency using a shortest MEP latency, which the relative frequency of MEP latency in pre-training period was firstly above 1%. We subtracted other MEP latency with the shortest MEP latency. Figure 4A shows histograms of the MEP latency data recorded before and after the training period in both sessions. Three-way repeated measures ANOVA yielded significant second-order interaction (session × time × latency: *F*_(20,200)_ = 1.67, *p* = 0.04, partial *η*^2^ = 0.14). Hence, we analyzed the data separately. In the implicit session, two-way repeated measures ANOVA detected a significant main effect of latency (*F*_(20,200)_ = 15.62, *p* < 0.01, partial *η*^2^ = 0.61), but not a main effect of time (pre-post: *F*_(1,10)_ = 4.34, *p* = 0.06, partial *η*^2^ = 0.30), and the interaction between these parameters was also significant (*F*_(20,200)_ = 2.50, *p* < 0.01, partial *η*^2^ = 0.20). In short-latency MEPs, significant reductions in the relative frequency were observed. In contrast, the relative frequency in long-latency MEPs was significantly increased. However, no such differences were seen in the explicit session, except for the main effect of latency (latency: *F*_(20,200)_ = 19.51, *p* < 0.01, partial *η*^2^ = 0.66, time: *F*_(1,10)_ = 2.63, *p* = 0.14, partial *η*^2^ = 0.21, interaction: *F*_(20,200)_ = 0.39, *p* = 0.99, partial *η*^2^ = 0.04). Figure 4B shows histograms of the MEP amplitude. No differences between the pre- and post-training data were detected in either session. In addition, the mean MEP amplitude and the number of MEPs of >0.25 mV remained unchanged in both sessions (Table 1).

**Table 1:** **Summary of motor-evoked potentials (MEP) measurements**.

	Implicit session		Explicit session	
	Pre	Post	Pre	Post
Resting threshold (% MSO)	45.18 ± 1.79	−	46.55 ± 2.04	−
rmsEMG of pre-trigger EMG (μV)	5.50 ± 0.21	5.69 ± 0.27	5.61 ± 0.19	5.44 ± 0.10
MEP amplitude (mV)	1.06 ± 0.19	1.09 ± 0.24	1.03 ± 0.17	1.19 ± 0.23
Number of MEPs of >0.25 mV	171.45 ± 12.30	160.27 ± 14.44	166.64 ± 11.07	171.72 ± 14.39
Mean MEP latency across 25 sites (ms)	22.17 ± 0.42*	22.53 ± 0.50	22.03 ± 0.33	22.10 ± 0.32

MSO, maximum stimulator output; rmsEMG, root mean squared EMG. Mean ± SE. *Implicit, pre vs. post, p < 0.05.

### Additional Experiment

Through the first experiment, we found the increases in MEP latency without any changes in MEP amplitude after implicit, but not explicit session. In the first experiment, the number of repetition of sequence trials differed between the two session. There remains a possibility that the different number of trials during training between the two sessions would affect the different changes in MEP measure. Therefore, we performed an additional experiment. Table 2 shows a summary of the result of additional experiment. As with the first experiment, the RTs during sequence trials were shortened through the training while the RTs during random trials did not change. For the MEP measure, we found no significant differences in MEP amplitude, MEP latency, and bEMG between the pre- and post-test.

**Table 2:** **Summary of additional experiment**.

	pre	post	*t* value	*p* value	*r* value
RT (random trials; ms)	414.11 ± 12.34	386.51 ± 16.86	1.55	0.17	0.51
RT (sequence trials; ms)	287.61 ± 35.65	180.02 ± 44.16	6.31	<0.01*	0.92
MEP amplitude (mV)	0.96 ± 0.15	1.01 ± 0.16	1.82	0.11	0.57
MEP latency (ms)	22.61 ± 0.41	22.53 ± 0.37	1.38	0.21	0.46
rmsEMG of pre-trigger EMG (μV)	5.61 ± 0.15	5.55 ± 0.17	0.70	0.50	0.26
Number of correctly recalled items	−	12.38 ± 1.55	−	−	

Mean ± SE. paired *t*-test, *pre vs. post.

## Discussion

The novelty of this study is the increases in MEP latency without the alteration of the orientation of the coil, the background EMG level, and the MEP amplitude through the implicit, but not the explicit session. Our findings suggest that the acquisition of implicit knowledge involves the reorganization of the corticomotor pathway. In contrast, acquiring explicit knowledge does not appear to induce the reorganization based on the measures we recorded.

### Reorganization of the Corticospinal System during the Acquisition of Implicit Knowledge

The memory systems associated with implicit and explicit knowledge are considered to be different (Pascual-Leone et al., 1994; Honda et al., 1998; Kantak et al., 2012; Tunovic et al., 2014). A previous study demonstrated that activity in the frontoparietal region was correlated with a parameter of explicit learning, and activity in the contralateral sensorimotor cortex was correlated with RT during the implicit learning phase (Honda et al., 1998). The results from transcranial direct current stimulation studies (Nitsche et al., 2003; Kantak et al., 2012) revealed that the M1 is involved in the acquisition of implicit knowledge. However, it still remains unclear whether the acquisition of implicit knowledge induces plastic changes in neuronal circuits in the M1. Pascual-Leone et al. (1994) reported increases in MEP map area and amplitude after implicit learning. By contrast, Tunovic et al. (2014) reported that implicit learning did not enhance MEP amplitude. Therefore, we examined the plasticity of the M1 through implicit and explicit learning by analyzing both the MEP amplitude and the MEP latency. In the present study, neither the amplitude nor latency of MEP changed during the explicit session. In contrast, we detected increases in MEP latency without any alterations in MEP amplitude during the implicit session, which indicates the reorganization of the corticomotor pathway, especially in the M1. The present finding suggests that plastic changes in the M1 do not necessarily induce modulation of MEP amplitude. In other words, only measuring MEP amplitude sometimes failed to capture plasticity in the M1 through motor learning. As described in the introduction, the MEP amplitude does not necessarily reflect the reorganization of the M1 as balanced excitatory and inhibitory plasticity in the M1, balanced M1 and spinal plasticity, and dissociation between the stimulation site and the site where plastic changes occur can all result in a lack of variation in MEP amplitude. Therefore, we proposed that analyzing a combination of MEP amplitude, MEP latency and/or other index such as joint kinematics is encouraged in order to capture plasticity of the motor cortices.

### Possible Mechanisms Responsible for the Observed Increases in MEP Latency

In the present study, we found the increases in MEP latency through implicit, but not explicit learning. Although it is impossible to know the detailed mechanisms of present results through our experiment, we describe some possibilities to explain the results.

In the implicit session, the significant bins of relative frequency of MEP latency were ~1.2–1.4 ms intervals. TMS induces I-waves that the intervals between the I-waves are about 1–1.5 ms-long (Day et al., 1989; Sakai et al., 1997; Di Lazzaro et al., 2008). The similarity between the intervals of the significant bins of MEP latency detected in the present study and the I-wave interval suggests modulation of I-wave components on motoneurons firing through training in the implicit, but not the explicit session. It was demonstrated that horizontal neuronal connections in the M1 are a strong candidate substrate for the reorganization of the M1 by motor learning (Sanes and Donoghue, 2000). Therefore, one possible mechanism is that horizontal neural circuits, which generate the I-waves, would be reorganized by the learning. Another possibility is that the increases in MEP latency would be coming from some other regions away from the M1, such as the premotor cortex. In a review article (Di Lazzaro et al., 2008), premotor cortex has been demonstrated to facilitate neuronal circuits in the M1 that generate later I-waves. In the present study, mapping procedure was used, which indicates that the premotor cortex would be stimulated. Hence, there remains a possibility that the effects of premotor cortex on later I-waves were enhanced through the implicit learning. In addition, changes in intrinsic property of early I-wave component, such as rise time of EPSP, would cause the MEP latency modulation without any changes in MEP amplitude.

### Limitations

In this study, we used higher stimulus intensity to evoke MEP than those previous studies. Furthermore, we did not examine the learning-induced plasticity using recruitment curve of MEP amplitude. This may cause no changes in MEP amplitude through the implicit session because of two reasons. One is that high intensity stimulation would stimulate other areas adjacent to the M1. The other is a ceiling effect. However, the results of previous studies (Rosenkranz et al., 2007; Rogasch et al., 2009) that investigated the changes in M1 excitability induced by motor training based on MEP recruitment curves suggest that a motor learning had similar or stronger effects on MEP amplitude when a high stimulus intensity was used than when a low stimulus intensity was employed. Although we could not rule out these possibilities, our use of a high stimulus intensity compared with those employed in previous studies would not affect our results. And also, we did not rule out a possibility for the changes in spinal motoneuron excitability through the training. The possibility will be investigated in future study.

In addition, there remains a possibility that our results were task-specific. Previous study has demonstrated the task-specific effects of direct current stimulation over the M1 on learning and memory formation (Saucedo Marquez et al., 2013). We do not know how the MEP latency and amplitude change through other type of implicit learning task (e.g., probability learning or adaptation to gradually alternating environment) and explicit learning task (e.g., sequence tapping task). Further studies that investigate the possibility are needed.

## Conclusion

The findings of this study suggest that the acquisition of implicit knowledge involves M1 reorganization. The changes in MEP latency with no alteration of MEP amplitude suggest balanced plastic changes at different neural substrates. Therefore, analyzing a combination of some indices of cortical excitability, such as MEP amplitude or latency, with mapping procedure is encouraged to fully understand plasticity across the motor cortices.

## Author Contributions

MH designed the study and analyzed data. All authors conducted the experiment and participated in the interpretation of the data. MH drafted the manuscript. SK and KF helped with the writing of the manuscript. All authors approved the final version of the manuscript submitted for publication.

## Conflict of Interest Statement

The authors declare that the research was conducted in the absence of any commercial or financial relationships that could be construed as a potential conflict of interest.
